# Follow-up investigation of antibody titers and diagnostic antibody cutoff values in patients with scrub typhus in South Korea

**DOI:** 10.1186/s12879-020-05735-8

**Published:** 2021-01-13

**Authors:** Choon-Mee Kim, Dong-Min Kim, Na Ra Yun

**Affiliations:** 1 Premedical Science, College of Medicine, Chosun University, Gwangju, Republic of Korea; 2 Departments of Internal Medicine, College of Medicine, Chosun University, Gwangju, Republic of Korea

**Keywords:** Scrub typhus, Fluorescent antibody technique, *Orientia tsutsugamushi*

## Abstract

**Background:**

Scrub typhus is a mite-borne infectious disease caused by *Orientia tsutsugamushi*. Few follow-up studies have assessed antibody titers using serologic tests from various commercial laboratories and the Korea Centers for Disease Control and Prevention (KCDC).

**Methods:**

A prospective study to assess the antibody titers in patients with scrub typhus and seroprevalence in individuals undergoing health checkups was conducted using results of immunofluorescence antibody assays (IFAs) and serologic tests, used by the KCDC and commercial laboratories, respectively. The following tests were performed simultaneously: (i) indirect IFA used by the KCDC to detect immunoglobulin (Ig) M and IgG, (ii) IFA used by a commercial laboratory to detect total Ig, and (iii) antibody tests using two commercially available kits.

**Results:**

When the IgM and IgG cutoff values (≥1:16 and ≥1:256, respectively) used in the IFA and the total IgG cutoff values (≥1:40) were used in prospective follow-up investigations, the antibody positivity rates of 102 patients with scrub typhus were 44.1, 35.3, and 57.6%, respectively, within 5 days of symptom onset. Among 91 individuals who recovered from scrub typhus, the follow-up IgM, IgG, and total Ig positivity rates for 13 years were 37.4% (34/91), 22.0% (20/91), and 76.9% (70/91), respectively. Among 216 individuals undergoing health checkups, the seroprevalence of IgM was 4.2% (9/216); no seroprevalence of IgG was observed.

**Conclusions:**

IFAs used by the KCDC and the commercial laboratory and rapid commercial kits could not distinguish between patients who had recovered from scrub typhus and those who are currently infected with *O. tsutsugamushi*. In South Korea and other countries, where low antibody cutoff values are used, upward adjustments of cutoff values may be necessary.

**Supplementary Information:**

The online version contains supplementary material available at 10.1186/s12879-020-05735-8.

## Background

Scrub typhus is a vector-borne infectious disease caused by *Orientia tsutsugamushi,* which is transmitted by a chigger bite [1]. The most common method of diagnosing scrub typhus is serological tests; indirect immunofluorescence antibody assays (IFA), enzyme-linked immunosorbent assays, and passive hemagglutination assays are used to detect the presence of antibodies (Abs) against *O. tsutsugamushi*. However, these tests have limited clinical usefulness because antibodies are usually formed 1–2 weeks after infection [2]. The gold standard diagnostic method is based on a ≥4-fold increase in Ab titer; however, the possibility of false-positive results cannot be excluded in cases with a 4-fold increase from a low titer [3]. Moreover, it is clinically difficult to perform follow-up investigations to check Ab titers. Even if Ab titers are monitored, a ≥4-fold increase may not be observed in cases with high early titers. In a previous study, a ≥4-fold increase in immunoglobulin (Ig) M and IgG levels were observed in only 61 and 72% cases, respectively, ≥1 month after symptom onset [4]. Hence, many countries use cutoff values to diagnose scrub typhus.

To identify an appropriate cutoff value of Ab titer in diagnostic serologic tests for scrub typhus, it is necessary to examine the titer levels and the duration for which these levels are sustained in patients who have recovered from scrub typhus. However, follow-up studies to examine titers in patients with scrub typhus or an assessment of diagnostic Ab cutoff values have rarely been performed [4].

In the present study, we conducted a follow-up investigation to examine Ab titers in patients with scrub typhus using IFA, which were used by the Korea Centers for Disease Control and Prevention (KCDC), and serologic tests, which were used by various commercial laboratories.

## Methods

This study included adults aged ≥18 years who had a history of fever within the past month, those who visited Chosun University Hospital between May 2016 and May 2018, and those who were diagnosed with scrub typhus. Ab titers were prospectively assayed every 7 days for 1 month after treatment and every 6 months for 18 months thereafter. The diagnostic criteria for scrub typhus were a ≥4-fold increase in levels of IgM or IgG Ab titers against *O. tsutsugamushi* in the IFA or positive results of nested polymerase chain reaction (PCR) targeting *O. tsutsugamushi*-specific 56-kDa gene or 16S rRNA gene [5, 6]. In addition, patients were examined for diseases similar to scrub typhus, including murine typhus, leptospirosis, hemorrhagic fever with renal syndrome, and systemic lupus erythematosus. Patients with evidence of these diseases were excluded to eliminate their potential confounding effects on the clinical manifestations and test results [1]. In addition to the prospective investigation, patients who were admitted to Chosun University Hospital and diagnosed with scrub typhus between 2004 and 2016 were contacted individually; those who provided consent underwent follow-up titer investigations. Serum samples were also obtained from individuals who underwent blood tests as part of the health checkup performed in the hospital during the study period.

Diagnostic tests using the KCDC method and Ab tests from various commercial laboratories were performed simultaneously. The indirect IFA used by the KCDC detects serum IgM and IgG in patients who respond to mixed *O. tsutsugamushi* antigens—Gilliam, Karp, Kato, and Boryoung—as described previously [4]. To perform IFA according to the KCDC method, two-fold serial dilutions from 1:16 human sera were reacted with mixed *O. tsutsugamushi* antigens. The IFA positive cutoff value for scrub typhus used by the KCDC was ≥1:16 for IgM and ≥1:256 for IgG.

The IFA for detecting total Ig was performed at a company in South Korea (designated as “A”), which is a commercial laboratory. This IFA measures the total Ab (IgM/IgG/IgA) against *O. tsutsugamushi* (Boryoung, Karp, and Gilliam antigens). Two-fold serial dilutions from 1:40 human sera were reacted with mixed *O. tsutsugamushi* antigens for Ab tests to detect total Ig by company A. The IFA positive cutoff value for scrub typhus used by company A was ≥1:40 for total Ig.

Immunochromatography was performed in the our hospital laboratory using two commercially available kits from companies designated as “C” and “D” in South Korea in accordance with manufacturers’ instructions. The commercial kit of company C is a diagnostic immunochromatography kit that uses a mixed antigen consisting of the 56-kDa protein of *O. tsutsugamushi* Kangwon and 21-kDa protein of *O. tsutsugamushi* Boryoung [7, 8]. The commercial kit of company D measures the total Ab (IgM/IgG/IgA) in serum samples using *O. tsutsugamushi* Gilliam, Karp, and Kato antigens [7].

## Results

**Fig. 1:**
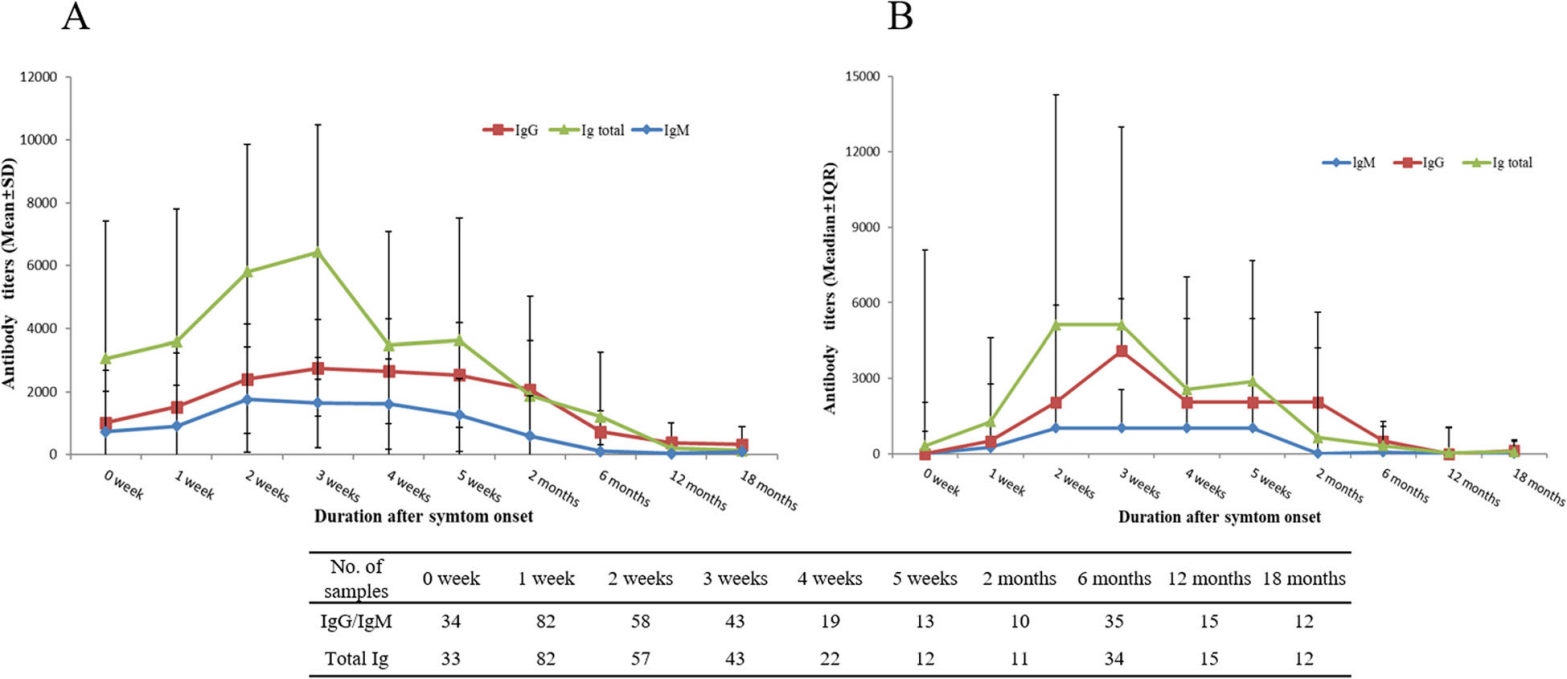
Prospective follow-up of antibody titers in patients diagnosed with scrub typhus. The antibody titers were measured by an immunofluorescence assay (IFA). **a**. Mean (± standard deviation) antibody titers. **b**. Median (± interquartile range) antibody titers. Data show total immunoglobulin (Ig) levels measured by company A, which is a commercial laboratory, and IgM/G titers measured by the IFA used by the KCDC. Cf>IFA IgM and IgG were checked in 34 serum samples, but total Ig was checked in 33 serum samples at 0 weeks.

**Fig. 2:**
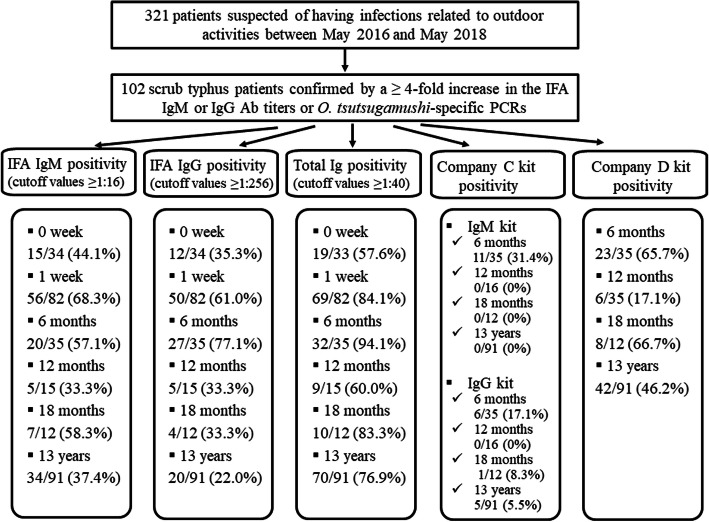
Overview of prospective and retrospective follow-up antibody titers determined by the following tests: (i) IFA used by KCDC to detect IgM and IgG, (ii) IFA used by company A to detect total Ig, and (iii) antibody tests using two commercially available kits from companies C and D

Of the 321 patients suspected of having infections related to outdoor activities between May 2016 and May 2018, 158 were suspected to have scrub typhus; 102 patients were confirmed to have *O. tsutsugamushi* infection (Supplementary Table 1). We followed up these patients by investigating their Ab titers. In total, 101 samples from 102 patients were sent to company A for Ab tests to detect the total Ig. The IgM titer values remained at peak for 2–5 weeks after symptom onset and subsequently decreased. Similarly, the IgG Ab titer values also rapidly increased 1–3 weeks after symptom onset, reached a peak at around 3–4 weeks, subsequently decreased and remained stable for 2 months, and finally declined slowly (Fig. 1). The total Ig Ab titer values rapidly increased around 1 week after symptom onset, reached a peak at 2–3 weeks, and subsequently declined (Supplementary Table 2). The Ab positivity rates within 5 days of symptom onset (0 week) were 44.1, 35.3, and 57.6% for cutoff values of ≥1:16 for IgM, ≥1:256 for IgG, and ≥1:40 for total Ig, respectively. The Ab positive rates were 68.3, 61.0, and 84.1%, respectively, within 6–10 days of symptom onset (1 week) (Supplementary Tables 1 and 2).The commercial kit of company C was used to investigate the serum samples of 35 patients who were followed up for approximately 6 months. Of the 35 patients, 11 were positive for IgM, corresponding to a positivity rate of 31.4%, and six were positive for IgG, corresponding to a positivity rate of 17.1%. When the commercial kit of company D was used, 23 of 35 patients showed positive results, corresponding to a positivity rate of 65.7% at approximately 6 months. None of the patients who were followed up for 18 months were positive for IgM; however, one patient was positive for IgG using the commercial kit of company C. Eight of 12 patients showed positive results, corresponding to a positivity rate of 66.7% at 18 months (Table 1) using the commercial kit of company D. A follow-up study regarding Ab titers was performed in healthy individuals who had recovered from scrub typhus. Overall, 636 patients were admitted to Chosun University Hospital for scrub typhus and were diagnosed positive for *O. tsutsugamushi* using PCR. Of these, the Ab titers of 91 individuals were followed up for ≥1 year and up to a maximum of 13 years after recovery; 34 (37.4%) had IgM titers ≥1:16. Of the 52 individuals who had recovered from scrub typhus in the past 5 years, 27 (51.9%) had IgM titers ≥1:16. Of the 28 individuals who were followed up for ≥6 years and up to 10 years, four (14.3%) had IgM titers ≥1:16. Of the 17 individuals who had recovered for > 10 years, three (17.6%) had IgM titers ≥1:16. In a 1-year period, one of 16 individuals with a IgM cutoff titer ≥1:128 had positive test results, corresponding to a positivity rate of 6.3%. None of the individuals had IgM titers ≥1:128 after 4 years. During the total follow-up period, 20 of the 91 (22.0%) individuals had IgG cutoff titers ≥1:256. IgG titers ≥1:256 were observed in 13 (25.0%) of 52 individuals who had recovered from scrub typhus in the last 5 years and six (21.4%) of 28 individuals who were followed up for ≥6 and up to 10 years. Of the 17 individuals who had recovered for > 10 years, two (11.8%) had IgG titers ≥1:256. Five of 91 individuals had IgG titers ≥1:1024, corresponding to a positivity rate of 5.5% (Table 2). Of the 91 patients for whom a total Ig titer values were investigated using the commercial kit of company A, 70 (76.9%) had total Ig titers ≥1:40 (Table 3). When the commercial kits of company C were used in 91 individuals who had recovered from scrub typhus, the samples were negative for IgM. Five (5.5%) of 91 individuals who were followed up for 13 years showed positive results for IgG. When the commercial kits of company D were used, 42 (46.2%) of 91 individuals whose Ab titers were followed up for 13 years showed a positive result (Table 4). To investigate seroprevalence, IFA was performed using samples from 216 individuals who underwent health checkups. When the cutoff value was set to ≥1:16 for IgM titers, 4.2% (9/216) individuals showed IgM seropositivity. However, when the cutoff value was set to 1:256 for IgG, seropositivity was absent (Table 5, Fig. 2). When the commercial kits of the companies C and D were used for samples from the 216 health checkup recipients, one patient showed positive results and the remaining 215 showed negative results using the commercial kits of company D; however, all 216 individuals were negative for IgM and IgG using the commercial kits of company C (data not shown).

**Table 1:** Prospective follow-up of antibody titers in patients with scrub typhus using rapid diagnostic test using immunochromatography performed using commercial kits from companies C and D

	6 Months			12 Months			18 Months		
	C company kit		D company kit	C company kit		D company kit	C company kit		D company kit
	IgM *n* (CP)	IgG *n* (CP)	*n* (CP)	IgM *n* (CP)	IgG *n* (CP)	*n* (CP)	IgM *n* (CP)	IgG *n* (CP)	*n* (CP)
Negative *n*	24 (68.6)	29 (82.9)	12 (34.3)	16 (100)	16 (100)	2 (12.5)	12 (100)	11 (91.7)	4 (33.3)
Positive *n*	11 (31.4)	6 (17.1)	23 (65.7)	0 (**0**)	0 (**0**)	14 (87.5)	0 (0)	1 (8.3)	8 (66.7)
Total *n*	35	35	35	16	16	16	12	12	12

*CP* Cumulative percentage

**Table 2:** Antibody titers in patients who had recovered from scrub typhus (*N* = 91) based on IgM and IgG positivity at several timepoints, as determined by the IFA used by the KCDC

IFA of KCDC	1 year		2 years		3 years		4 years		5 years		6 years		7 years		8 years		9 years		10 years		11 years		12 years		13 years	
	IgM *n* *(CP)*	IgG *n* *(CP)*	IgM *n* *(CP)*	IgG *n* *(CP)*	IgM *n* *(CP)*	IgG *n* *(CP)*	IgM *n* *(CP)*	IgG *n* *(CP)*	IgM *n* *(CP)*	IgG *n* *(CP)*	IgM *n* *(CP)*	IgG *n* *(CP)*	IgM *n* *(CP)*	IgG *n* *(CP)*	IgM *n* *(CP)*	IgG *n* *(CP)*	IgM *n* *(CP)*	IgG *n* *(CP)*	IgM *n* *(CP)*	IgG *n* *(CP)*	IgM *n* *(CP)*	IgG *n* *(CP)*	IgM *n* *(CP)*	IgG *n* *(CP)*	IgM *n* *(CP)*	IgG *n* *(CP)*
**0**	10 (100)	10 (100)	4 (100)	2 (100)	6 (100)	5 (100)	1 (100)	1 (100)	4 (100)	2 (100)	6 (100)	3 (100)	3 (100)	1 (100)	3 (100)	2 (100)	6 (100)	3 (100)	6 (100)	2 (100)	3 (100)	3 (100)	2 (100)	1 (100)	3 (100)	2 (100)
**1:16**	**1** (**37.5**)	0 (37.5)	**0** (**50**)	0 (75)	**2** (**66.7**)	0 (72.2)	**1** (**66.7**)	0 (66.7)	**1** (**42.9**)	0 (71.4)	**0** (**25**)	0 (62.5)	**2** (**40**)	0 (80)	0 (0)	0 (33.3)	0 (0)	0 (50)	0 (0)	1 (66.7)	**1** (**50**)	0 (50)	0 (0)	0 (50)	0 (0)	0 (33.3)
**1:32**	3 (31.3)	0 (37.5)	2 (50)	1 (75)	5 (55.6)	5 (72.2)	1 (33.3)	0 (66.7)	0 (28.6)	0 (71.5)	1 (25)	0 (62.5)	0 (0)	1 (80)	0 (0)	0 (33.3)	0 (0)	1 (50)	0 (0)	0 (50)	0 (33.3)	0 (50)	0 (0)	0 (50)	0 (0)	1 (33.3)
**1:64**	1 (12.5)	0 (37.5)	1 (25)	1 (62.5)	3 (27.8)	2 (44.4)	0 (0)	0 (66.7)	2 (28.6)	2 (71.4)	1 (12.5)	1 (62.5)	0 (0)	2 (60)	0 (0)	1 (33.3)	0 (0)	1 (33.3)	0 (0)	1 (50)	2 (33.3)	0 (50)	0 (0)	0 (50)	0 (0)	0 (0)
**1:128**	0 (6.3)	0 (37.5)	1 (12.5)	1 (50)	2 (11.1)	4 (33.3)	0 (0)	1 (66.7)	0 (0)	2 (42.9)	0 (0)	1 (50)	0 (0)	0 (20)	0 (0)	0 (0)	0 (0)	0 (16.7)	0 (0)	1 (33.3)	0 (0)	2 (50)	0 (0)	1 (50)	0 (0	0 (0)
**1:256**	1 (6.3)	**1** (**37.5**)	0 (0)	**2** (**37.5**)	0 (0)	**1** (**11.1**)	0 (0)	**1** (**33.3**)	0 (0)	**0** (**14.3**)	0 (0)	**2** (**37.5**)	0 (0)	**0** (**20**)	0 (0)	**0** (**0**)	0 (0)	**0** (**16.7**)	0 (0)	**1** (**16.7**)	0 (0)	**1** (**16.7**)	0 (0)	**0** (**0**)	0 (0)	**0** (**0**)
**1:512**	0 (0)	1 (31.3)	0 (0)	1 (12.5)	0 (0)	1 (5.6)	0 (0)	0 (0)	0 (0)	1 (14.3)	0 (0)	1 (12.5)	0 (0)	1 (20)	0 (0)	0 (0)	0 (0)	0 (16.7)	0 (0)	0 (0)	0 (0)	0 (0)	0 (0)	0 (0)	0 (0)	0 (0)
**1:1024**	0 (0)	3 (25)	0 (0)	0 (0)	0 (0)	0 (0)	0 (0)	0 (0)	0 (0)	0 (0)	0 (0)	0 (0)	0 (0)	0 (0)	0 (0)	0 (0)	0 (0)	1 (16.7)	0 (0)	0 (0)	0 (0)	0 (0)	0 (0)	0 (0)	0 (0)	0 (0)
**1:2048**	0 (0)	1 (6.3)	0 (0)	0 (0)	0 (0)	0 (0)	0 (0)	0 (0)	0 (0)	0 (0)	(0 (0)	0 (0)	0 (0)	0 (0)	0 (0)	0 (0)	0 (0)	0 (0)	0 (0)	0) (0	0 (0)	0 (0)	0 (0)	0 (0)	0 (0)	0 (0)
**Total** ***n***	16		8		18		3		7		8		5		3		6		6		6		2		3	

Bold indicates the results obtained at cutoff values of ≥1:16 for IgM and ≥1:256 for IgG, which are reference cutoff values for positivity

*CP* cumulative percentage, *IFA* immunofluorescence assay, *KCDC* Korean Centers for Disease Control and Prevention

**Table 3:** Antibody titers in patients who had recovered from scrub typhus (*N* = 91) based on positive rates of the total antibody titer, as determined by the IFA used by company A

IFA of A Company	1 year	2 years	3 years	4 years	5 years	6 years	7 years	8 years	9 years	10 years	11 years	12 years	13 years
	n (CP)	n (CP)	n (CP)	n (CP	n (CP)	n (CP)	n (CP)	n (CP	n (CP)	n (CP)	n (CP)	n (CP)	n (CP)
**0**	6 (100)	0 (100)	2 (100)	1 (100)	1 (100)	1 (100)	1 (100)	1 (100)	1 (100)	1 (100)	3 (100)	2 (100)	1 (100)
**1:40**	**3 (62.5)**	**0 (100)**	**8 (88.9)**	**0 (66.7)**	**1 (85.7)**	**4 (87.5)**	**2 (80)**	**1 (66.7)**	**4 (83.3)**	**1 (83.3)**	**0 (50)**	**0 (0)**	**2 (66.7)**
**1:80**	1 (43.8)	0 (100)	1 (44.4)	0 (66.7)	3 (71.4)	0 (37.5)	1 (40)	1 (33.3)	0 (16.6)	1 (66.7)	3 (50)	0 (0)	0 (0)
**1:160**	1 (37.5)	2 (100)	4 (38.9)	1 (66.7)	2 (28.6)	3 (37.5)	1 (20)	0 (0)	0 (16.6)	3 (50.0)	0 (0)	0 (0)	0 (0)
**1:320**	2 (31.3)	2 (75.0)	0 (16.7)	1 (33.3)	0 (0)	0 (0)	0 (0)	0 (0)	0 (16.6)	0 (0)	0 (0)	0 (0)	0 (0)
**1:640**	2 (18.8)	4 (50.0)	3 (16.7)	0 (0)	0 (0)	0 (0)	0 (0)	0 (0)	0 (16.6)	0 (0)	0 (0)	0 (0)	0 (0)
**1:1280**	1 (6.3)	0 (0)	0 (0)	0 (0)	0 (0)	0 (0)	0 (0)	0 (0)	0 (16.6)	0 (0)	0 (0)	0 (0)	0 (0)
**1:2560**	0 (0)	0 (0)	0 (0)	0 (0)	0 (0)	0 (0)	0 (0)	0 (0)	0 (16.6)	0 (0)	0 (0)	0 (0)	0 (0)
**1:5120**	0 (0)	0 (0)	0 (0)	0 (0)	0 (0)	0 (0)	0 (0)	0 (0)	1 (16.6)	0 (0)	0 (0)	0 (0)	0 (0)
**> 1:5120**	0 (0)	0 (0)	0 (0)	0 (0)	0 (0)	0 (0)	0 (0)	0 (0)	0 (0)	0 (0)	0 (0)	0 (0)	0(0)
**Total n**	16	8	18	3	7	8	5	3	6	6	6	2	3

Bold indicates the results obtained at cutoff values of ≥1:40 for total Ig detection, which are reference cutoff values for positivity

*CP* Cumulative percentage, *IFA* Immunofluorescence assay

**Table 4:** Antibody titers in patients who had recovered from scrub typhus (*N* = 91) based on follow-up at several timepoints using commercial kits from companies C and D

	Company	Antibody	Negative ***n***	Positive ***n***	Total ***n***
**1 year**	C	IgM	16	0	16
		IgG	16	0	
	D		2	14	
**2 years**	C	IgM	8	0	8
		IgG	7	1	
	D		2	6	
**3 years**	C	IgM	18	0	18
		IgG	18	0	
	D		10	8	
**4 years**	C	IgM	3	0	3
		IgG	3	0	
	D		2	1	
**5 years**	C	IgM	7	0	7
		IgG	7	0	
	D		3	4	
**6 years**	C	IgM	8	0	8
		IgG	8	0	
	D		7	1	
**7 years**	C	IgM	5	0	5
		IgG	5	0	
	D		5	0	
**8 years**	C	IgM	3	0	3
		IgG	3	0	
	D		3	0	
**9 years**	C	IgM	6	0	6
		IgG	2	4	
	D		3	3	
**10 years**	C	IgM	6	0	6
		IgG	6	0	
	D		6	0	
**11 years**	C	IgM	6	0	6
		IgG	6	0	
	D		3	3	
**12 years**	C	IgM	2	0	2
		IgG	2	0	
	D		1	1	
**13 years**	C	IgM	3	0	3
		IgG	3	0	
	D		2	1	

C, C company kit; D, D company kit

**Table 5:** IgM/G positivity determined by the IFA used by the KCDC in individuals undergoing health checkup

	IgM	IgG
	*n (CP)*	*n (CP)*
0	207 (100)	179 (100)
1:16	9 (4.2)	16 (17.1)
1:32	0 (0)	15 (9.7)
1:64	0 (0)	3 (2.8)
1:128	0 (0)	3 (1.4)
Total	216	216

*CP* Cumulative percentage, *IFA* Immunofluorescence assay, *KCDC* Korean Centers for Disease Control and Prevention

## Discussion

This prospective study to assess the antibody titers in 102 patients with scrub typhus showed that the antibody positivity rates within 5 days of symptom onset were 44.1, 35.3, and 57.6% when cutoff values were used ≥1:16 for IgM, ≥1:256 for IgG, and ≥1:40 for total Ig, respectively. The IgM, IgG, and total Ig positivity rates for 13 years in the follow-up investigations with 91 individuals who recovered from scrub typhus were 37.4, 22.0, and 76.9%, respectively.

A limitation of serological tests for the diagnosis of scrub typhus is the insufficient formation of antibodies in the early or acute phases of infection, which could lead to false-negative results. To set an appropriate cutoff for the region and for accurate diagnosis, it is necessary to have information regarding seroprevalence, Ab titers in the endemic region, and Ab titers maintained in patients who have recovered from scrub typhus. Otherwise, false-positive results may be obtained. Moreover, in an endemic situation, using Ab tests to detect total Ig may lead to more frequent false-positive results as a consequence of IgG remaining from a previous infection [8].

According to a study by Blacksell on the usefulness of the indirect immunofluorescence method, which is the gold standard for diagnosis of scrub typhus [9], the cutoff value used in Thailand and Malaysia (range, 1:50–1:400) differs from that used in Japan and South Korea (range, 1:10–1:40). The most commonly used single titer cutoff value was 1:400. Many countries, including Thailand and Malaysia, use a cutoff value as high as 1:400. In this prospective study, most subjects (14/15, 93.3%) underwent negative seroconversion within 1 year when a cutoff value ≥1:128 was used. However, lower cutoff values (1:10–1:40), such as those used in Korea and Japan, resulted in six of 16 patients showing positive result at 1 year (37.5%) when the IgM value was set at 1:16 in our study.

To accurately diagnose scrub typhus, negative diagnostic test results need to be observed in patients who have recovered from past scrub typhus. Therefore, it is important to know how long Ab levels are maintained in patients who have recovered. Moreover, to evaluate the accuracy of the various IFAs, which are employed in commercial laboratories, patients who have recovered from scrub typhus should be studied, and the false-positive results in this population should be determined for each assay kit.

The KCDC’s criteria for scrub typhus reporting and diagnosis are cutoff values ≥1:16 for IgM and ≥1:256 for IgG. In this study, Ab titers were evaluated in 91 individuals who had recovered from scrub typhus and were followed up for a maximum of 13 years. In the IFA used by company A to measure total Ig for 13 years, 76.9% individuals showed a positive result. Using the commercial kits of company D, 46.2% individuals showed a positive result within 13 years. When IgM and IgG cutoff values (≥1:16 and ≥1:256, respectively) used in the KCDC’s IFA were applied within 13 years, 37.3 and 22.0%, respectively, patients showed positive results. These results show that IFAs and commercial kits of company D are highly likely to misdiagnose diseases other than scrub typhus as patients who previously had scrub typhus showed positive results due to the presence of lingering residual Abs.

We also evaluated the test results of 91 healthy individuals who had recovered from scrub typhus and who were followed up for 13 years to set the IFA IgM cutoff. When the IgM cutoff was set to 1:128, four (4.4%) of 91 individuals showed a positive result when the test was conducted within 13 years and four (5%) of 80 individuals showed a positive result when the test was conducted within 10 years. None of the individuals showed a positive result after 10 years. When the IgG cutoff was set to 1:1024 in the IFA, five (5.5%) of 91 individuals showed a positive result within 13 years and six (7.5%) of 80 individuals showed a positive result within 10 years. None of the individuals showed a positive result after 10 years. Therefore, to distinguish patients with previous infection from those with current infection, an upward adjustment of cutoff values is needed, including those for IFAs performed by the KCDC and company A.

Since there are statistically significant differences in the seroprevalence of scrub typhus across geographical regions, studies on the baseline seroprevalence in each region are important to determine a diagnostic cutoff value for scrub typhus [10]. An Ab titer analysis was performed among healthy individuals who underwent blood tests as part of a health checkup in a university hospital. This analysis showed that the IgM seropositivity rate was 4.2% (9/216) for a cutoff value of ≥1:16 and the IgG seropositivity was absent for a cutoff value of 1:256. Therefore, an IgM cutoff value of 1:16 meant that 4.2% healthy individuals are likely to be misdiagnosed with scrub typhus; thus, an upward adjustment of the IgM titer is necessary.

The limitation of this study is that the data were obtained from patients who visited a single university hospital in Gwangju for medical examination; they do not reflect the background Ab titer of the entire country. The national seroprevalence and background Ab titers must be considered when determining the cutoff value for diagnosing scrub typhus. In addition to considerations regarding Ab titer longevity in previously infected patients, data on Ab cross-reactions in patients with other acute infections must also be reviewed to determine the cutoff value. Thus, prospective studies on cross-reactions and diagnostic accuracy are needed to determine an appropriate cutoff value for each country. Another limitation of this study is that all samples from patients with scrub typhus who visited the hospital were collected periodically and then frozen at − 20 °C. We performed diagnostic tests to assess the antibody titers for scrub typhus at the same time or at a similar time.

## Conclusion

In conclusion, this study shows that the commercial kits commonly used in clinical settings in South Korea and IFA used by the KCDC could not distinguish patients who had recovered from scrub typhus from those who currently have scrub typhus. In South Korea and other countries, where cutoff values for titers are used as the criteria for reporting and diagnosing scrub typhus, an upward adjustment of the cutoff values may be necessary. To determine an appropriate cutoff value, prospective studies on background Ab titers are needed at a national level.

## Supplementary Information

**Additional file 1: Table S1.** Prospective follow-up of antibody titers in patients with scrub typhus (*N* = 102) based on IgM/G antibody titers determined by the IFA method used by the KCDC. **Table S2.** Prospective follow-up of antibody titers in patients with scrub typhus (N = 101) based on total IgG antibody titers determined by the IFA used by company A (a commercial laboratory).

## Data Availability

The datasets used and/or analysed during the current study are available from the corresponding author on reasonable request.

## Abbreviations

KCDC: Korea Centers for Disease Control and Prevention;
IFA: Immunofluorescence antibody assay
Ab: Antibody
Ig: Immunoglobulin
PCR: Polymerase chain reaction
