# Supplementary material for: Follow-up investigation of antibody titers and diagnostic antibody cutoff values in patients with scrub typhus in South Korea
Source: BMC Infect Dis. 2021 Jan 13;21:69. doi: 10.1186/s12879-020-05735-8 (PMC7807423; doi:10.1186/s12879-020-05735-8)
Supplement: Supplementary file 1 — Additional file 1: Table S1. Prospective follow-up of antibody titers in patients with scrub typhus (N = 102) based on IgM/G antibody titers determined by the IFA method used by the KCDC. Table S2. Prospective follow-up of antibody titers in patients with scrub typhus (N = 101) based on total IgG antibody titers determined by the IFA used by company A (a commercial laboratory). [file 12879_2020_5735_MOESM1_ESM.docx]

| Supplementary Table 1. Prospective follow-up of antibody titers in patients with scrub typhus (N = 102) based on IgM/G antibody titers determined by the IFA method used by the KCDC | | | | | | | | | | | | | | | | | | |  | | |
| --- | --- | --- | --- | --- | --- | --- | --- | --- | --- | --- | --- | --- | --- | --- | --- | --- | --- | --- | --- | --- | --- |
| IFA of KCDC | **0 weeks** | | **1 week** | | **2 weeks** | | **3 weeks** | | **4 weeks** | | **5 weeks** | | **2 months** | | **6 months** | | **12 months** | | **18 months** | | |
|  | IgM　 n  (CP) | IgG　 n  (CP) | IgM　 n  (CP) | IgG　 n  (CP) | IgM　 n  (CP) | IgG　 n  (CP) | IgM　 n  (CP) | IgG　 n  (CP) | IgM　 n  (CP) | IgG　 n  (CP) | IgM　 n  (CP) | IgG　 n  (CP) | IgM　 n  (CP) | IgG　 n  (CP) | IgM　 n  (CP) | IgG　 n  (CP) | IgM　 n  (CP) | IgG　 n  (CP) | IgM　 n  (CP) | IgG　 n  (CP) |  |
| **0** | 19  (100) | 22  (100) | 26  (100) | 21  (100) | 13  (100) | 11  (100) | 4  (100) | 5  (100) | 0  (100) | 0  (100) | 1  (100) | 0  (100) | 5  (100) | 0  (100) | 15  (100) | 6  (100) | 10  (100) | 10  (100) | 5  (100) | 3  (100) |  |
| **1:16** | 0  (44.1) | 0  (35.3) | 3  (68.3) | 3  (80.3) | 0  (78.9) | 0  (85.2) | 0  (90.7) | 0  (88.4) | 0  (100) | 0  (100) | 0  (92.3) | 0  (100) | 0  (50) | 0  (100) | 3  (57.1) | 0  (82.9) | 1  (33.3) | 0  (33.3) | 2  (58.3) | 0  (75.0) |  |
| **1:32** | 0  (44.1) | 0  (35.3) | 5  (64.5) | 1  (76.3) | 0  (78.9) | 0  (85.2) | 0  (90.7) | 0  (88.4) | 0  (100) | 0  (100) | 0  (92.3) | 0  (100) | 1  (50) | 0  (100) | 2  (48.6) | 1  (82.9) | 2  (26.7) | 0  (33.3) | 4  (41.6) | 0  (75.0) |  |
| **1:64** | 1  (44.1) | 0  (35.3) | 3  (58.5) | 4  (75.0) | 0  (78.9) | 0  (85.2) | 0  (90.7) | 0  (88.4) | 0  (100) | 0  (100) | 0  (92.3) | 0  (100) | 0  (40) | 0  (100) | 4  (42.9) | 0  (80.0) | 1  (13.3) | 0  (33.3) | 1  (8.3) | 2  (75.0) |  |
| **1:128** | 1  (41.2) | 0  (35.3) | 1  (54.9) | 3  (70.0) | 3  (78.9) | 0  (85.2) | 1  (90.7) | 0  (88.4) | 1  (100) | 1  (100) | 0  (92.3) | 1  (100) | 0  (40) | 1  (100) | 4  (31.4) | 1  (80.0) | 0  (6.7) | 0  (33.3) | 0  (0) | 3  (58.3) |  |
| **1:256** | 3  (38.2) | 1  (35.3) | 10  (53.7) | 5  (65.8) | 3  (73.7) | 4  (85.2) | 5  (88.4) | 1  (88.4) | 1  (94.7) | 0  (94.7) | 2  (92.3) | 0  (92.3) | 1  (40) | 0  (90) | 5  (20.0) | 5  (77.1) | 1  (6.7) | 0  (33.3) | 0  (0) | 1  (33.3) |  |
| **1:512** | 2  (29.4) | 1  (32.4) | 5  (41.5) | 5  (59.2) | 6  (68.4) | 2  (77.8) | 4  (76.7) | 0  (86.0) | 5  (89.5) | 4  (94.7) | 3  (76.9) | 2  (92.3) | 1  (30) | 2  (90) | 1  (5.7) | 9  (62.9) | 0  (0) | 1  (33.3) | 0  (0) | 2  (25.0) |  |
| **1:1024** | 0  (23.5) | 1  (29.4) | 11  (35.4) | 7  (52.6) | 5  (57.9) | 2  (74.1) | 10  (67.4) | 3  (86.0) | 5  (63.2) | 1  (73.7) | 2  (53.8) | 1  (76.9) | 1  (20) | 1  (70) | 1  (2.9) | 7  (37.1) | 0  (0) | 3  (26.7) | 0  (0) | 0  (8.3) |  |
| **1:2048** | 5  (23.5) | 2  (26.5) | 8  (22.0) | 11  (43.4) | 11  (49.1) | 12  (70.4) | 10  (44.2) | 12  (79.1) | 3  (36.8) | 3  (68.4) | 4  (38.5) | 3  (69.2) | 0  (20) | 3  (60) | 0  (0) | 6  (17.1) | 0  (0) | 1  (6.7) | 0  (0) | 1  (8.3) |  |
| **>1:2048** | 3  (8.8) | 7  (20.6) | 10  (12.2) | 22  (28.9) | 17  (29.8) | 27  (48.1) | 9  (20.9) | 22  (51.2) | 4  (21.1) | 10  (52.6) | 1  (7.6) | 6  (46.2) | 1  (10) | 3  (30) | 0  (0) | 0  (0) | 0  (0) | 0  (0) | 0  (0) | 0  (0) |  |
| **Total** | 34 | | 82 | | 58 | | 43 | | 19 | | 13 | | 10 | | 35 | | 15 | | 12 | | |
| CP = cumulative percentage; IFA = immunofluorescence assay; KCDC = Korea Centers for Disease Control and Prevention  Durations after onset: 0 weeks = 0–5 days, 1 week = 6*–*10 days, 2 weeks = 11*–*17 days, 3 weeks = 18*–*25 days, 4 weeks = 26*–*33 days, 5 weeks = 34*–*35 days | | | | | | | | | | | | | | | | | | |  | | |

Supplementary Table 2. Prospective follow-up of antibody titers in patients with scrub typhus (N = 101) based on total IgG antibody titers determined by the IFA used by company A (a commercial laboratory)

| **IFA**  **of A company** | **0 weeks** | **1 week** | **2 weeks** | **3 weeks** | **4 weeks** | **5 weeks** | | **2 months** | **6 months** | **12 months** | **18 months** | |  |
| --- | --- | --- | --- | --- | --- | --- | --- | --- | --- | --- | --- | --- | --- |
|  | n  (CP) | n  (CP) | n  (CP) | n  (CP) | n  (CP) | n  (CP) | n  (CP) | | n  (CP) | n  (CP) | n  CP) | |  |
| **0** | 14  (100) | 13  (100) | 1  (100) | 0  (100) | 0  (100) | 0  (100) | 0  (100) | | 2  (100) | 6  (100) | 2  (100) | |  |
| **1:40** | **1**  (**57.6**) | **6**  (**84.1**) | **0**  (**98.2**) | **0**  (**100**) | **0**  (**95.5**) | **0**  (**100**) | **0**  (**100**) | | **2**  (**94.1**) | **3**  (**60.0**) | **3**  (**83.3**) | |  |
| **1:80** | 1  (54.5) | 1  (76.8) | 0  (98.2) | 0  (100) | 1  (95.5) | 0  (100) | 0  (100) | | 1  (88.2) | 1  (40.0) | 2  (58.3) | |  |
| **1:160** | 0  (51.5) | 11  (75.6) | 3  (93.0) | 1  (100) | 1  (90.9) | 2  (100) | 2  (100) | | 9  (85.3) | 1  (40.0) | 4  (41.7) | |  |
| **1:320** | 2  (51.5) | 2  (62.2) | 3  (91.7) | 0  (97.7) | 0  (86.4) | 2  (84.6) | 1  (81.8) | | 4  (58.8) | 2  (33.3) | 0  (8.3) | |  |
| **1:640** | 0  (45.5) | 7  (59.8) | 3  (87.7) | 4  (97.7) | 4  (86.4) | 2  (69.2) | 5  (72.7) | | 3  (47.1) | 1  (26.7) | 1  (8.3) | |  |
| **1:1280** | 3  (45.5) | 3  (51.2) | 5  (82.4) | 5  (88.4) | 3  (72.7) | 0  (53.8) | 1  (27.3) | | 8  (38.2) | 1  (13.3) | 0  (0) | |  |
| **1:2560** | 3  (36.4) | 9  (47.6) | 4  (73.7) | 3  (76.7) | 7  (59.1) | 0  (46.2) | 0  (27.3) | | 2  (14.7) | 0  (0) | 0  (0) | |  |
| **1:5120** | 1  (27.3) | 10  (36.6) | 16  (66.7) | 10  (69.8) | 2  (27.3) | 4  (46.2) | 1  (18.2) | | 2  (8.8) | 0  (0) | 0  (0) | |  |
| **>1:5120** | 8  (24.2) | 20  (24.4) | 22  (38.6) | 20  (46.5) | 4  (18.2) | 2  (15.4) | 1  (9.1) | | 1  (2.9) | 0  (0) | 0  (0) | |  |
| **Total**  **n** | 33 | 82 | 57 | 43 | 22 | 12 | 11 | | 34 | 15 | 12 | |  |
| Bold indicates the results obtained at cutoff values of ≥1:40 for total Ig detection, which are reference cutoff values for positivity.  CP = cumulative percentage; IFA = immunofluorescence assay  Durations after onset: 0 weeks = 0–5 days, 1 week = 6*–*10 days, 2 weeks = 11*–*17 days, 3 weeks = 18*–*25 days, 4 weeks = 26*–*3 days, 5 weeks = 34*–*35 days | | | | | | | | | | | |  | |
